# What Constitutes a High‐Quality Guideline: Exploring Consumers' Views

**DOI:** 10.1002/ueg2.70000

**Published:** 2025-02-28

**Authors:** Alberto Balduzzi, Francesco Maria Carrano, Yngve Falck‐Ytter, Haluk Tarik Kani, Iris Levink, Yasuko Maeda, Irene Marafini, Adele Sayers, EAES/ECCO/ESCP/ESGAR/ESGE/ESPGHAN/UEG/UEGJ Quality of Guideline Collaborative Group

**Affiliations:** ^1^ General Surgery Unit Pederzoli Hospital Verona Italy; ^2^ Department of Medical and Surgical Sciences and Translational Medicine Faculty of Medicine and Psychology St Andrea Hospital Sapienza University Rome Italy; ^3^ Gastroenterology and Hepatology Department of Medicine Case Western Reserve University VA Northeast Ohio Healthcare System Cleveland Ohio USA; ^4^ Department of Gastroenterology Marmara University School of Medicine Istanbul Turkey; ^5^ Institute of Gastroenterology Marmara University Istanbul Turkey; ^6^ Department of Gastroenterology and Hepatology Erasmus MC Medical Centre Rotterdam the Netherlands; ^7^ Department of Surgery Queen Elizabeth University Hospital and University of Glasgow Glasgow UK; ^8^ Department of Systems Medicine Gastroenterology Unit Department of Medical Science University of Rome Tor Vergata Tor Vergata University Hospital Rome Italy; ^9^ Sheffield Teaching Hospitals NHS Foundation Trust and University of Sheffield Sheffield UK

**Keywords:** AGREE‐II, development, EBM, evidence‐based medicine, GRADE, guidelines, improvement, methodology, recommendations, systematic review

## Abstract

**Introduction:**

Clinical guidelines are a cornerstone of evidence‐based medicine. Little is known about clinicians' knowledge of guideline development and how they perceive guideline quality.

**Methods:**

A survey protocol was designed according to the CHERRIES (improving the quality of web surveys: the Checklist for Reporting Results of Internet E‐Surveys) checklist. The survey explored three main aspects: high‐quality markers of guidelines, knowledge of guideline development, and areas for improvement. The survey was conducted by contacting UEG and affiliated societies by email and via social media. All valid answers to each question were counted.

**Results:**

A total of 585 participants responded during the 3‐month period. Some 65.8% were aged between 30 and 60 years, and 75.4% were doctors. The most important perceived quality indicators within a guideline were ‘clear and actionable recommendations (97%)’, followed by ‘based on systematic literature review’ (96%), and ‘transparent methodology’ (90%). 230 (39.3%) respondents were previously involved in clinical guideline development. However, the experience of working with a methodologist (18.8%) and using well‐established guideline checklists (AGREE‐II [21.0%]), RIGHT (Reporting Items for Practice guidelines in HealThcare) (9.9%) were limited. Just under half of the responders (289, 49.4%) were familiar with the GRADE methodology. Apps (78.5%), webinars (73.8%), and short videos (68.2%) were popular tools to access clinical guidelines. Over 90% of responders stated that the reputation of the journal (92%) and the name of the society involved in guideline development (91%) were important. Two‐thirds of the responders preferred to see abridged versions of guidelines and 69.2% preferred freely accessible or open access guidelines.

**Conclusion:**

Consumers are keen to read clear and actionable guidelines that are developed transparently. There is a gap in guideline development knowledge. Initiatives by medical journals and professional societies are important to ensure the development of accessible and robust clinical guidelines.

## Graphical abstract

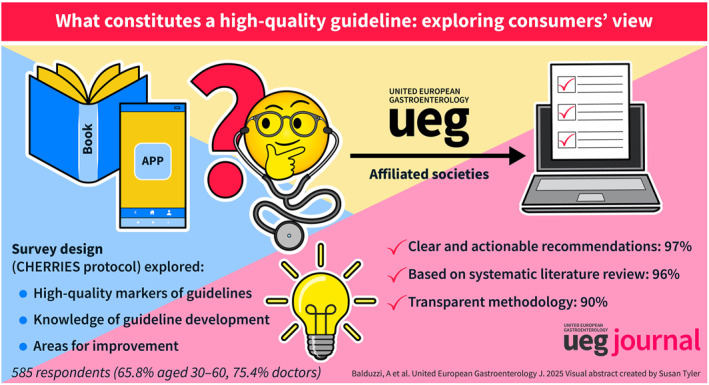

1

**Summary**

- Summarise the established knowledge on this subject
  - Initiatives by medical journals and professional societies are important to ensure accessible and robust clinical guidelines development using GRADE and adherence to its methodology.
- What are the significant and/or new findings of this study?
  - Consumers are keen to read clear and actionable guidelines that are developed transparently.
  - There is a gap in guideline development knowledge.

## 2 Background

Evidence‐based medicine has become the cornerstone of high‐quality clinical care for patients. At its inception in the 1990s, much of the emphasis was placed on extracting high‐quality evidence from randomised controlled trials. This process has evolved over the last three decades and is much more nuanced in its interpretation.

The Grading of Recommendations Assessment, Development and Evaluation (GRADE) is a well‐established and transparent system to develop evidence‐based guidelines. During the last 2 decades, it has been adopted by more than 100 organizations, including the World Health Organization [1].

However, many guidelines in gastroenterology and gastrointestinal surgery are still produced without a transparent evidence assessment and decision‐making process [2, 3, 4, 5]. Evidence is often ranked solely on the basis of study type. Recommendations are often made by collating expert opinions or without a clear explanation of background rationale. In addition, lack of consideration for the acceptability, cost, patient's values and preferences and equity in the recommendations, coupled with failure to reflect patient‐centred outcome, result in dogmatic or impractical guidelines that are difficult to implement.

Little is known about consumers’ (clinicians, healthcare professionals, stakeholders and patients) understanding of guideline development and what would be the ideal platform and format for sharing guidelines. A so‐called ‘consumer survey’ of guidelines has not been carried out systematically and this is important for shaping the future of guideline publication.

A survey was designed to assess readers’ knowledge of guideline development, the unmet needs for quality guidelines, and how we can drive quality improvement.

## 3 Methods

### 3.1 Design

This survey was intended to target gastroenterologists, gastro‐intestinal surgeons, general practitioners, radiologists, and other clinicians with an interest within the field of gastroenterology, trainees, physician associates, nurse practitioners, nurses, nutritionists, researchers, industry personnel and patients interested in GI disorders.

We aimed to address three key questions:

1. What are high‐quality guidelines according to consumers?
  To assess the level of awareness and familiarity among GI healthcare professionals and consumers regarding key concepts that are required to develop and critically appraise clinical guidelines.
2. What is the current knowledge level of guideline development in the GI community?
  To understand if clinicians are familiar with the modern guideline development standards such as GRADE and to understand what users focus on when they select and read a guideline, apart from the relevance of the topic to their daily practice. The factors explored included author names, journal selection, practical algorithms, assessment calculators, up‐to‐date knowledge, and being user‐ or reader‐friendly.
3. How can we work towards improving the quality of guidelines?
  To collect suggestions and recommendations from participants on what additional format they prefer for guidelines, such as shorter versions, an infographic, a series of snapshots of a guideline, a guideline webinar or a podcast.

### 3.2 Ethical Considerations and Data Protection

As this was a consumer survey, ethical approval or informed consent was not needed. However, consumers were notified that participation was voluntary and that only anonymised data would be published. No incentives were offered for participation. All collected data were recorded in Qualtrics at the University of Glasgow and were stored anonymously and non‐identifiable. However, an IP‐check was performed to prevent duplicates.

### 3.3 Survey Development

The study protocol was designed based on the CHERRIES (improving the quality of web surveys: the Checklist for Reporting Results of Internet E‐Surveys) checklist. The survey was developed by steering group members who were familiar with the guideline development process. During a separate study assessing the quality of published guidelines using the AGREE‐II tool and qualitative interviews of guideline developers and clinicians, several themes emerged, which were reflected in the survey [6]. Adaptive questioning was used to reduce the number and complexity of questions. The number of questions per page was restricted to 5 to increase the engagement rate. Usability and functionality were tested by the steering group prior to opening the link to participants.

### 3.4 Survey Dissemination and Data Collection

The survey was disseminated to participants for 3 months. The link was disseminated by the participating UEG's international societies to its memberships (please see Acknowledgement) and was further cascaded to other national societies and via social media.

### 3.5 Statistical Analysis

All data were exported from Qualtrics to an Excel sheet. Numbers are described in absolute count or % of all responses including no answers for demographics. For other questions, all completed or answered responses were used as denominators. Categorical comparisons were performed using Fisher’s exact test, and *p*‐values of < 0.05 were deemed statistically significant.

## 4 Results

### 4.1 Demographics

A total of 585 participants responded to the survey between 20th March and 4th June 2024. Two‐thirds of respondents were in their 30s (159, 27.2%), 40s (127, 21.7%) and 50s (99, 16.9%). 233 (39.8%) respondents were physicians in a gastroenterology or surgery department, and 208 (35.6%) were physicians in another speciality. Some 93 (15.9%) responded as ‘other’, 36 (6.2%) were health care allied professionals, and 15 (2.6%) were general practitioners. Nearly three‐quarters of the respondents were specialists or completed training (421, 72.0%), whilst 72 (12.3%) were in training. Almost two‐thirds of the respondents (384, 65.6%) were involved in research activities. The majority of respondents were from Europe 393 (67.2%). The demographic data were summarised in Table 1.

**TABLE 1:** Demographics of survey respondents.

	Total cohort (*n* = 585)
Age, *n* (%)	
20–29 years	26 (4.4)
30–39 years	159 (27.2)
40–49 years	127 (21.7)
50–59 years	99 (16.9)
> 60 years	80 (13.7)
Not answered	94 (16.1)
Type of responder, *n* (%)	
Gastroenterologist/Surgeon	233 (39.8)
Physician in other specialities	208 (35.6)
General practitioners	15 (2.6)
Medical student and other students	6 (1.0)
Nurse/physiotherapist/occupational therapist/nutritionist (without prescribing rights)	6 (1.0)
Physician assistant/advanced nurse practitioner/pharmacists (with prescribing rights)	4 (0.7)
Other consumers who do not belong to any of the above	20 (3.4)
Not answered	93 (15.9)
Training status, *n* (%)	
Not in training	421 (72.0)
In training	72 (12.3)
Not answered	92 (15.7)
Does research activities in daily practice, *n* (%)	
No	108 (18.5)
Yes	384 (65.6)
Not answered	93 (15.9)
Continent of responders, *n* (%)	
Africa	11 (1.9)
Asia	43 (7.4)
Australia/Oceania	12 (2.1)
Europe	393 (67.2)
North America	13 (2.2)
Latin America	16 (2.7)
Not answered	97 (16.6)

### 4.2 Experience of Guideline Development

**FIGURE 1:**
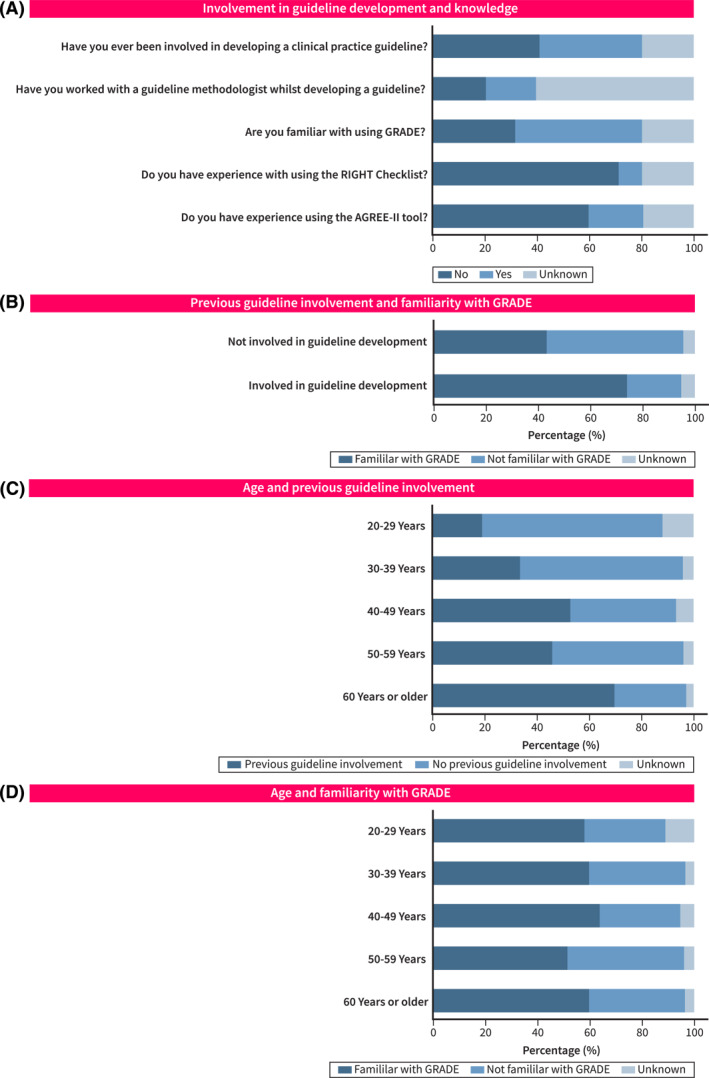
Involvement in guideline development (A), knowledge (A and B), and their relationship with age (C and D).

230 (39.3%) respondents were previously involved in clinical guideline development, 241 (41.1%) were not; 114 (19.5%) respondents did not answer this question (Figure 1A). Experience of working with a methodologist while developing a guideline was limited to 18.8% (110) of all respondents or 47.8% (110 of 230) of those who previously developed a guideline.

The experience of using well‐established guideline checklists was also limited. Only 123 (21.0%) and 58 (9.9%) of all responders had experience of using AGREE‐II (347 [59.3%]) and RIGHT (Reporting Items for Practice guidelines in HealThcare) checklists, respectively. Just under half of the responders (289, 49.4%) were familiar with the GRADE methodology. Those who were previously involved in guideline development were more often familiar with GRADE (*p* < 0.001; Figure 1B). Two‐thirds of respondents between 20 and 39 years old (118 of 176, 67%) had not been involved in guideline development before, compared to those above age 40 who had experience (58.2%, 170 of 292, *p* < 0.05; Figure 1C). However, familiarity with GRADE was not associated with age (*p* > 0.05, Figure 1D).

**FIGURE 2:**
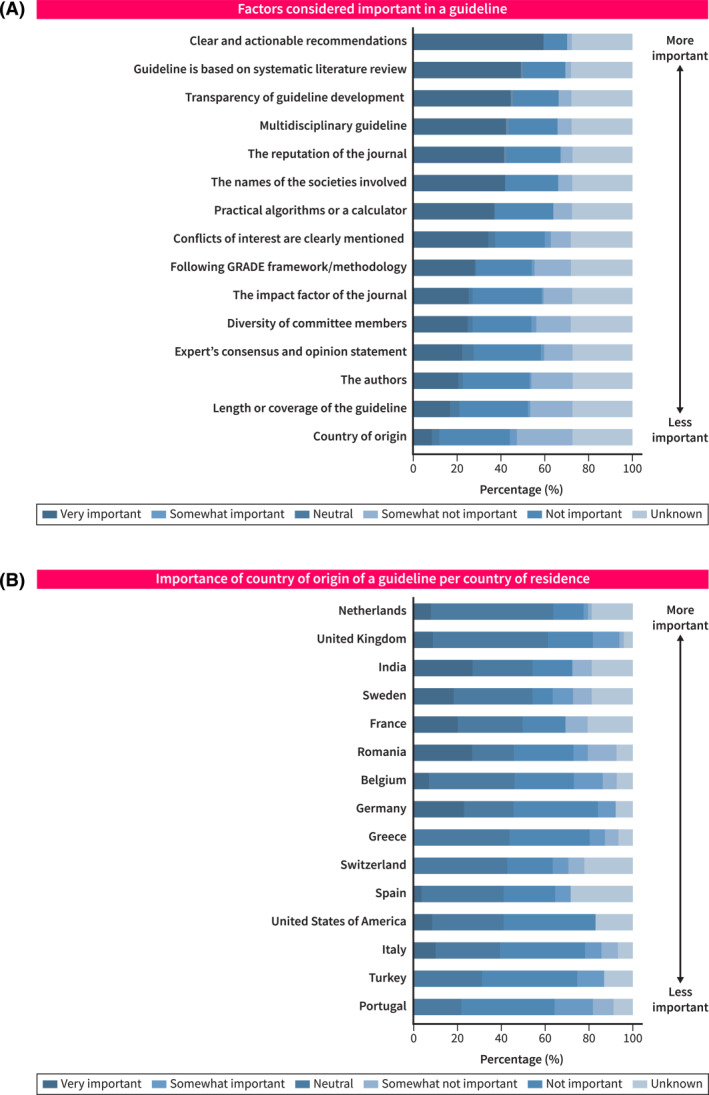
Factors considered important in a guideline (A) and importance of country of origin of a guideline per country of residence (B).

The most frequently cited components deemed to be of significant importance to responders were ‘clear and actionable recommendations’ (411 of 425 responders, 97%), a ‘systematic literature review’ (407 of 424, 96%), and ‘transparent methodology’ (383 of 424, 90%) (Figure 2A). The country of origin was deemed to be of some degree of importance by 229 of the 425 (53.8%, Figure 2A). Responders from the Netherlands, the United Kingdom and India considered the country of origin to be of significant importance (Figure 2B).

**FIGURE 3:**
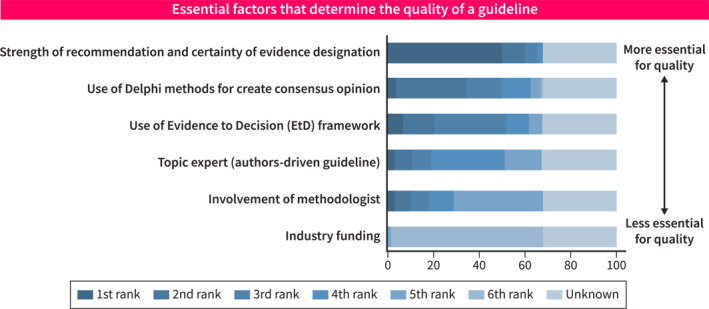
Factors that determine the quality of a guideline.

Strength of recommendation and certainty of evidence were chosen as the top factors to determine the quality of guidelines by 299 (74.9%) respondents. The use of Delphi methods and the Evidence to Decision framework were second and third factors to be benchmarks for the quality of guidelines (Figure 3).

**FIGURE 4:**
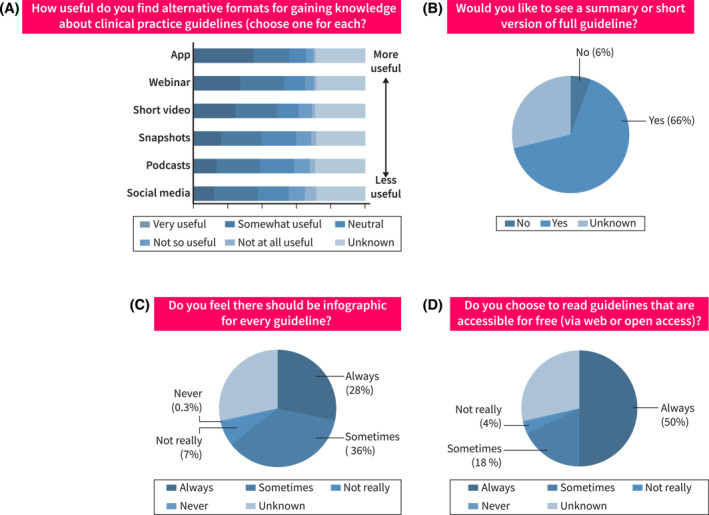
Alternative formats were found useful for gaining knowledge about clinical guidelines.

The format for accessing clinical practice guidelines was evaluated across different age groups (Figure 4A, Figure S1). The most useful formats across all ages were apps, webinars, and short videos. Guideline apps were rated as highly useful, with over 60% of respondents in each age group indicating that they found them to be either ‘very useful’ or ‘somewhat useful’. The utility of social media platforms was generally perceived to be low, particularly among older age groups. Podcasts demonstrated a relatively consistent level of perceived utility across age ranges, with approximately 40%–50% of respondents in each group rating them as ‘very useful’ or ‘somewhat useful’.

Additionally, the study investigated preferences regarding the format and accessibility of clinical practice guidelines (Figure 4B–D). Two‐thirds (66%) of the respondents preferred the provision of summary or abridged versions of comprehensive guidelines. Twenty‐eight percent of participants expressed the view that infographics should always accompany guidelines, while 36% indicated that they should be included occasionally. Accessibility was identified as a significant factor, with 50% of respondents preferring guidelines accessible via the internet for free or open access.

### 4.3 Summary of Free Text Entries

The respondents were encouraged to add their thoughts and comments at the end of the survey. There were comments on variable topics, most commonly on guideline principles and development. For example, one respondent stated ‘Guidelines can lose credibility when the strength of recommendation exceeds the clarity of message from the data. Expert‐opinion‐led‐recommendations can reinforce dogma.’ This sentiment was echoed by many, such as ‘People’s expectations from guidelines—strong recommendations for practice, are not always possible from the evidence. I strongly believe that guidelines should be evidence‐based and the methodology transparent, as well as assumptions about resources and settings.’

Some also touched upon the challenges of guideline development, for example, ‘Anyone who has been involved in guidelines knows that often decisions and votes skew from science. GRADE is often abused, and papers are not scored properly. On the other hand, the vast body of literature that is ballooning the findings makes assessing all available data very problematic and time‐consuming for busy physicians.’

Duplication and applicability of guidelines were also mentioned several times, such as ‘Some guidelines are issued as if there was a competition to publish them’, ‘there are far too many guidelines.’, ‘Guidelines are very defensive and create a lot of waste.’,’ They promote overdiagnosis and overtreatment. We live in a time with a limitation of resources; guidelines should help in making choices to cut out low value care and focus on a few essential items', ‘one‐size‐fits‐all guidelines (with legal repercussions if they are not fully met) to me are generally useless. Secondly, guidelines must guide in doubtful situations; too often they are used as directives/orders in routine practice and (mis)used to harass and intimidate colleagues.’

Many also commented on the importance of multi‐societal, multinational across‐the‐board guidelines and the diversity and inclusivity of guideline members with the involvement of patients and younger clinicians to reflect wider views and opinions. Some raised concerns that guidelines are often developed by resource‐rich country physicians or resource abundant specialist centres that may not be applicable to the rural/general clinical practice. A few commented on the need to be up to date with timely renewal of published guidelines. Other comments included the requirement for training to develop guidelines, and for guidelines to be open access to allow wider readership.

## 5 Discussion

This survey attempted to systematically assess the current understanding of the guideline development process by various stakeholders, including healthcare professionals, researchers, and patients. We also explored what readers perceive as quality benchmarks of clinical guidelines and unmet needs when developing clinical practice guidelines in gastroenterology and gastrointestinal surgery. The findings highlight several key areas for improvement in guideline development, dissemination, and implementation.

The survey results expose a significant knowledge gap among healthcare professionals regarding guideline development methodologies, even among those already involved in creating guidelines. Despite nearly half of those who answered the question saying they were involved in guideline development, not many were familiar with checklists such as AGREE II and RIGHT. Although nearly two‐thirds stated they were familiar with GRADE, not many have worked with a methodologist either. This reflects that the guidelines they were involved with either did not implement GRADE appropriately or they were involved in traditional guidelines of collating good practice statements, and were not stringently following development methodology. There is a clear division between guidelines based on evidence and good clinical practice, which is not necessarily based on evidence but reflecting common sense or acceptable practice [7].

This underscores the need for better education and dedicated training initiatives on established methodologies and tools, including the GRADE framework. There is now an established training program such as InGuide launched in September 2020 (https://inguide.org/). It is important that clinicians have sufficient knowledge of guideline development whilst soliciting help from expert methodologists and taking in opinions of wider stakeholders including accounting for different clinical settings, patients and considering the impact of recommendations on resources, cost and acceptability by patients or patients, it is important that clinical guidelines enable them to make informed decisions into their care, including detailed information of both the benefits and harms of all available opinions [8]. GRADE not only allows robust evidence assessment, but also works in tandem with the Evidence to Decision framework, allowing guideline developers to consider the aforementioned factors, ultimately leading to more reliable, accurate recommendations.

The survey results also show a preference for accessible guideline formats. Healthcare professionals favour short versions, infographics, and alternative media formats such as apps, webinars, and short videos. This suggests that traditional, lengthy guideline formats may not be effectively meeting the needs of modern users. It is pertinent that guideline developers create an efficient summary with key recommendations and use diverse formats to enhance dissemination and uptake. Age demographics also seem to play a role in format preferences. Younger respondents showed a higher preference for digital platforms such as guideline apps, while older groups favoured more traditional formats. This implies a potential generational gap and may indicate the need for diverse dissemination strategies to reach different age groups. Understanding demographic‐specific preferences is important to ensure that guidelines are accessible and engaging.

The reputation of the journals and authors as well as the societies involved in guideline development also significantly influence guideline selection among readers. Involving known reputable experts and institutions in guideline creation appears to be a feature looked for by the readers for credibility and trust of the material. However, the readers also expressed the importance of multidisciplinary and diverse members to be involved. Interestingly, the country of origin of a guideline, while not a primary factor, does hold some relevance for respondents. Guideline developers should be mindful of the potential impact of regional or national contexts on guideline applicability and consider tailoring recommendations to specific settings when appropriate. This practice could enhance guideline adoption and implementation.

Finally, the preference for free and open‐access guidelines emphasizes the importance of removing financial barriers to ensure equitable access.

This survey was widely disseminated among UEG and affiliated societies. This allowed us to receive responses from nearly 600 participants, which is a great strength of this project. However, one of the main study limitations was the inability to capture the total membership of the involved societies and calculate the number of people to whom the survey was disseminated to allow the proportion of responders to be determined. It is probable that the survey was answered by those with a vested interest in guideline production and may not have captured the views of those not interested in guideline methodology or development.

In conclusion, our survey provides valuable insights into the perspectives of healthcare professionals and relevant stakeholders on clinical practice guidelines in gastroenterology and gastrointestinal surgery. By addressing the identified knowledge gaps, listening to user preferences for concise and accessible formats, and prioritising methodological rigour and transparency in guideline development, the quality and impact of guidelines on clinical practice can be further improved.

## Ethics Statement

The authors have nothing to report.

## Conflicts of Interest

The authors declare no conflicts of interest.

## Supporting information

Supporting Information S1

Figure S1

Table S1

Table S2

## Data Availability

Data are available upon request.
