# Supplementary material for: What Constitutes a High‐Quality Guideline: Exploring Consumers' Views
Source: United European Gastroenterol J. 2025 Feb 28;13(2):268–75. doi: 10.1002/ueg2.70000 (PMC11975600; doi:10.1002/ueg2.70000)
Supplement: Supplementary file 1 — Supporting Information S1 [file UEG2-13-268-s001.docx]

# **Supplementary Document S1**

# Survey Draft

Thank you for considering taking part in this survey.

We are keen to explore what factors you look at when choosing a guideline and your understanding of how guidelines are developed.

It should take no more than 5-10 minutes to complete this survey.

Are you a physician, surgeon, other healthcare professional, academic/researcher, personnel at industry, or patient or family/carer of a patient interested in guidelines related to gastrointestinal disorders/diseases?

If yes, click to proceed

Otherwise, select “no” to come to a window to say: You are unable to participate. We thank you for your interest in the survey.

-------------------------------------------------------------------------------------------------------------------------------

I agree to take part [mandatory] [tick box]

*[If participants do not provide their informed consent, they can’t move to the next block and fulfil the questionnaire.]*

**About you:**

1. Which age group do you belong to?

1. 20-29
2. 30-39
3. 40-49
4. 50-59
5. >60

2. Please choose one from below that represent you most?

1. Gastroenterologist/Surgeon
2. Physician associates/physician assistant/advanced nurse practitioner/pharmacists (those with prescribing rights)
3. Nurse/physiotherapist/occupational therapist (those without prescribing rights)
4. General practitioners with GI interest
5. Physician in other speciality who have cross interest in GI diseases/disorders
6. Nutritionists/dietician
7. Industry personnel (eg exhibitor, employee of pharmaceutical company)
8. Medical student and other students associated with GI related degree
9. Patient, family/relative/carer of the patient
10. Other consumer who does not belong to any of the above (but uses, is affected by, GI guidelines): please specify

3. Are you in training?

1. yes
2. no

4. Do you do any research activity in your daily practice (e.g. participating or promoting

research projects, grant applications, writing papers...)?

1. yes
2. no

5. What is your country of residence?

[drop-down menu]

6. Which society are you mainly affiliated with (one choice only)?

1. AGA (American Gastroenterological Association)
2. ACG (American College of Gastroenterology)
3. ASGE (American Society for Gastrointestinal Endoscopy)
4. AASLD (American Association for the Study of Liver Diseases)

EAES ([1. Siemieniuk R, Guyatt G. What is GRADE? Available from: https://bestpractice.bmj.com/info/us/toolkit/learn-ebm/what-is-grade/](https://eaes.eu/)

[2. Gordon M, Guyatt G. Assessment of Evidence Quality in Inflammatory Bowel Disease Guidance: The Use and Misuse of GRADE. Gastroenterology. 2020 Oct;159(4):1209–15.](https://eaes.eu/)

[3. Wang X, Zheng MY, He HY, Zhu HL, Zhao YF, Chen YH, et al. Quality Evaluation of Guidelines for the Diagnosis and Treatment of Liver Failure. Critical Care Medicine [Internet]. 2024 Jun 4 [cited 2024 Sep 16]; Available from: https://journals.lww.com/10.1097/CCM.0000000000006346](https://eaes.eu/)

[4. Levink IJM, Balduzzi A, Marafini I, Kani HT, Maeda Y, UEGJ Guideline Taskforce. Quality of clinical guidelines: It matters as it impacts patient care. UEG Journal. 2024 Jul;12(6):664–6.](https://eaes.eu/)

[5. Guyatt GH, Schünemann HJ, Djulbegovic B, Akl EA. Guideline panels should not GRADE good practice statements. Journal of Clinical Epidemiology. 2015 May;68(5):597–600.](https://eaes.eu/)

1. y)
2. EAGEN (European Association for Gastroenterology, Endoscopy & Nutrition)
3. EASL (The European Association for the Study of the Liver )
4. ECCO (The European Crohn's and Colitis Organisation)
5. EDS (European Digestive Surgery)
6. EFISDS (European Federation International Society for Digestive Surgery)
7. EHMSG (European Helicobacter and Microbiota Study Group)
8. EPC (European Pancreatic Club)
9. ESCP (European Society of Coloproctology)
10. ESDO (the European Society of Digestive Oncology)
11. ESGAR (European Society of Gastrointestinal and Abdominal Radiology)
12. ESGE (The European Society of Gastrointestinal Endoscopy)
13. ESNM (The European Society of Neurogastroenterology and Motility)
14. ESP (European Society of Pathology)
15. ESPCG (European Society for Primary Care Gastroenterology)
16. ESPEN (The European Society for Clinical Nutrition and Metabolism)
17. ESPGHAN (The European Society for Paediatric Gastroenterology Hepatology and Nutrition)
18. UEG (United European Gastroenterology)
19. Others not on the list

**Knowledge of guideline development**

This section is intended to explore your knowledge about guideline development methodology.

7. Have you ever been involved in developing a clinical practice guideline?

1. Yes
2. No

8. Do you have experience using the AGREE-II tool for evaluating clinical practice guidelines?

1. Yes
2. No

9. Do you have experience using *RIGHT* (Reporting Items for practice *Guidelines* in

HealThcare) Checklist?

1. Yes
2. No

10. Are you familiar with GRADE (Grading of Recommendations, Assessment, Development, and Evaluations) methodology for grading the quality of evidence and strength of recommendations in guidelines?

1. Yes
2. No

11. Have you worked with a guideline methodologist whilst developing a guideline?

1. Yes
2. No

[Question 11 is only available if question 7 is ‘Yes’]

**Use of a guideline**

This section consists of 2 questions, exploring factors that matter to you when you choose to read a guideline and what you feel are essential ingredients of quality guidelines.

11. What are the factors that you consider important when selecting a clinical practice guideline to read (please choose the importance: very important, somewhat important, neutral, somewhat less important, Not very important, for each factor)?

1. The reputation of the journal
2. The impact factor of the journal
3. The names of authors (authors’ list)
4. The name of societies involved in the guideline
5. The country of origin
6. Committee members are from diverse background (eg different countries, centres)
7. Multidisciplinary guideline
8. Eye-catching infographics
9. Practical algorithms / calculators
10. Clear and actionable recommendations
11. Following GRADE framework/methodology
12. Transparency of guideline development
13. Based on systematic literature review
14. Based on experts’ consensus and opinion statement
15. Length or border coverage of guideline
16. Conflicts of interest management is clearly mentioned

Others [FREE TEXT]

12. What are the three essential factors that determine the quality of guidelines?

1. Strength of recommendation and certainty of evidence designation
2. Use of Delphi methods for create consensus opinion
3. Use of Evidence to Decision (EtD) framework
4. Topic expert authors-driven guideline
5. Involvement of methodologist
6. Appropriate industry collaboration

Others [FREE TEXT]

**Format of guideline**

This section asks you what format of guidelines you find helpful or would like to see in future.

13. Would you like to see a summary or short version of full guideline published in a

peer-reviewed journal?

- 1. Yes
  2. No

14. Do you feel there should be an infographic for every guideline?

1. Always
2. Sometimes
3. Not really
4. Never

15. How useful do you find alternative formats such as short videos, webinars, podcasts, snapshots, guideline app, social media dissemination, for gaining knowledge about clinical practice guidelines (choose one for each)?

|  | Very useful | Somewhat useful | Neutral | Somewhat useless | Not very useful |
| --- | --- | --- | --- | --- | --- |
| Short video |  |  |  |  |  |
| Webinar |  |  |  |  |  |
| Podcast |  |  |  |  |  |
| Snapshot |  |  |  |  |  |
| Guideline app |  |  |  |  |  |
| Social media |  |  |  |  |  |

**Access to the guideline**

16. Do you choose to read guidelines that are accessible for free (via web or open access)

compared to those behind a paywall?

1. Always
2. Sometimes
3. Not really
4. Never

17. Any other thoughts about the Guideline generation process [FREE TEXT]?

Email address :

I am happy to be contacted using the above email address [tick box]

I do not want to be contacted using the above email address [tick box]
