# Supplementary material for: What Constitutes a High‐Quality Guideline: Exploring Consumers' Views
Source: United European Gastroenterol J. 2025 Feb 28;13(2):268–75. doi: 10.1002/ueg2.70000 (PMC11975600; doi:10.1002/ueg2.70000)
Supplement: Supplementary file 2 — Figure S1 [file UEG2-13-268-s003.docx]

**Supplemental Figure S1. The association between the different alternative formats and age.**
