# Supplementary material for: What Constitutes a High‐Quality Guideline: Exploring Consumers' Views
Source: United European Gastroenterol J. 2025 Feb 28;13(2):268–75. doi: 10.1002/ueg2.70000 (PMC11975600; doi:10.1002/ueg2.70000)
Supplement: Supplementary file 3 — Table S1 [file UEG2-13-268-s002.docx]

**Supplementary Table S1: Country of residence of responders**

| **Country** | **Continent** | **Number** | **Percentage (%)** |
| --- | --- | --- | --- |
| Afghanistan | Asia | 1 | 0,2 |
| Albania | Europe | 1 | 0,2 |
| Argentina | Latin America | 4 | 0,7 |
| Armenia | Asia | 3 | 0,5 |
| Australia | Australia/oceania | 9 | 1,5 |
| Austria | Europe | 9 | 1,5 |
| Azerbaijan | Asia | 1 | 0,2 |
| Bahrain | Asia | 1 | 0,2 |
| Belgium | Europe | 15 | 2,6 |
| Bosnia and Herzegovina | Europe | 3 | 0,5 |
| Brazil | Latin America | 8 | 1,4 |
| Bulgaria | Europe | 5 | 0,9 |
| Canada | North America | 1 | 0,2 |
| Chile | Latin America | 1 | 0,2 |
| China | Asia | 1 | 0,2 |
| Croatia | Europe | 5 | 0,9 |
| Cyprus | Europe | 2 | 0,3 |
| Czech Republic | Europe | 3 | 0,5 |
| Denmark | Europe | 1 | 0,2 |
| Egypt | Africa | 5 | 0,9 |
| Estonia | Europe | 3 | 0,5 |
| Finland | Europe | 2 | 0,3 |
| France | Europe | 10 | 1,7 |
| Germany | Europe | 13 | 2,2 |
| Greece | Europe | 16 | 2,7 |
| India | Asia | 11 | 1,9 |
| Indonesia | Asia | 1 | 0,2 |
| Iran | Asia | 1 | 0,2 |
| Ireland | Europe | 6 | 1,0 |
| Israel | Asia | 5 | 0,9 |
| Italy | Europe | 66 | 11,3 |
| Japan | Asia | 4 | 0,7 |
| Latvia | Europe | 1 | 0,2 |
| Lebanon | Asia | 1 | 0,2 |
| Libyan Arab Jamahiriya | Africa | 1 | 0,2 |
| Lithuania | Europe | 3 | 0,5 |
| Macedonia | Europe | 1 | 0,2 |
| Malaysia | Asia | 1 | 0,2 |
| Malta | Europe | 9 | 1,5 |
| Mexico | Latin America | 1 | 0,2 |
| Netherlands | Europe | 50 | 8,5 |
| New Zealand | Australia/oceania | 3 | 0,5 |
| Nigeria | Africa | 1 | 0,2 |
| Norway | Europe | 4 | 0,7 |
| Paraguay | Latin America | 1 | 0,2 |
| Philippines | Asia | 1 | 0,2 |
| Poland | Europe | 6 | 1,0 |
| Portugal | Europe | 23 | 3,9 |
| Qatar | Asia | 1 | 0,2 |
| Romania | Europe | 15 | 2,6 |
| Russian Federation | Asia | 3 | 0,5 |
| Serbia | Europe | 5 | 0,9 |
| Singapore | Asia | 1 | 0,2 |
| Slovakia | Europe | 3 | 0,5 |
| Slovenia | Europe | 7 | 1,2 |
| South Korea | Asia | 3 | 0,5 |
| Spain | Europe | 29 | 5,0 |
| Sweden | Europe | 11 | 1,9 |
| Switzerland | Europe | 14 | 2,4 |
| Syrian Arab Republic | Asia | 2 | 0,3 |
| Tunisia | Africa | 1 | 0,2 |
| Turkey | Europe | 16 | 2,7 |
| Ukraine | Europe | 2 | 0,3 |
| United Arab Emirates | Africa | 2 | 0,3 |
| United Kingdom | Europe | 34 | 5,8 |
| United States of America | North America | 12 | 2,1 |
| Uruguay | Latin America | 1 | 0,2 |
| Viet Nam | Asia | 1 | 0,2 |
| Yemen | Africa | 1 | 0,2 |
| Unknown | Unknown | 97 | 16,6 |
