# Supplementary material for: What Constitutes a High‐Quality Guideline: Exploring Consumers' Views
Source: United European Gastroenterol J. 2025 Feb 28;13(2):268–75. doi: 10.1002/ueg2.70000 (PMC11975600; doi:10.1002/ueg2.70000)
Supplement: Supplementary file 4 — Table S2 [file UEG2-13-268-s004.docx]

Supplementary Table S2: Society membership of responders

| Society | Numbers | Percentage |
| --- | --- | --- |
| AASLD (American Association for the Study of Liver Diseases) | 2 | 0,3 |
| ACG (American College of Gastroenterology) | 4 | 0,7 |
| AGA (American Gastroenterological Association) | 7 | 1,2 |
| ASGE (American Society for Gastrointestinal Endoscopy) | 8 | 1,4 |
| EAES (The European Association of Endoscopic Surgery) | 47 | 8,0 |
| EAGEN (European Association for Gastroenterology, Endoscopy & Nutrition) | 2 | 0,3 |
| EASL (The European Association for the Study of the Liver ) | 11 | 1,9 |
| ECCO (The European Crohn's and Colitis Organisation) | 15 | 2,6 |
| EDS (European Digestive Surgery) | 1 | 0,2 |
| EHMSG (European Helicobacter and Microbiota Study Group) | 1 | 0,2 |
| EPC (European PancreaticClub) | 8 | 1,4 |
| ESCP (European Society of Coloproctology) | 23 | 3,9 |
| ESDO (the European Society of Digestive Oncology) | 10 | 1,7 |
| ESGAR (European Society of Gastrointestinal and Abdominal Radiology) | 178 | 30,4 |
| ESGE (The European Society of Gastrointestinal Endoscopy) | 20 | 3,4 |
| ESNM (The European Society of Neurogastroenterology and Motility) | 9 | 1,5 |
| ESP (European Society of Pathology) | 1 | 0,2 |
| ESPCG (European Society for Primary Care Gastroenterology) | 4 | 0,7 |
| ESPEN (The European Society for Clinical Nutrition and Metabolism) | 1 | 0,2 |
| ESPGHAN (The European Society for Paediatric Gastroenterology Hepatology and Nutrition) | 30 | 5,1 |
| Other society not on the list | 70 | 12,0 |
| UEG (United European Gastroenterology) | 29 | 5,0 |
| Unknown | 104 | 17,8 |
